# Autoinducer-2 regulates *Pseudomonas aeruginosa* PAO1 biofilm formation and virulence production in a dose-dependent manner

**DOI:** 10.1186/s12866-015-0529-y

**Published:** 2015-09-29

**Authors:** Hongdong Li, Xingyuan Li, Zhengli Wang, Yakun Fu, Qing Ai, Ying Dong, Jialin Yu

**Affiliations:** Department of Neonatology, Children’s Hospital, Chongqing Medical University, Chongqing, China; Ministry of Education Key Laboratory of Child Development and Disorders, Chongqing, China; Key Laboratory of Pediatrics in Chongqing and Chongqing International Science and Technology Cooperation Center for Child Development and Disorders, Chongqing, China; Department of Pharmacy, Chongqing Red Cross Hospital, Chongqing, China; Department of Paediatrics, Children’s Hospital of Fudan University, Shanghai, China

**Keywords:** Autoinducer-2, Quorum sensing, Biofilm, *Pseudomonas aeruginosa*

## Abstract

**Background:**

*Pseudomonas aeruginosa* is an opportunistic pathogen that is the leading cause of iatrogenic infections in critically ill patients, especially those undergoing mechanical ventilation. In this study, we investigated the effects of the universal signaling molecule autoinducer-2 (AI-2) in biofilm formation of *P. aeruginosa* PAO1.

**Results:**

The addition of 0.1 nM, 1 nM, and 10 nM exogenous AI-2 to *P. aeruginosa* PAO1 increased biofilm formation, bacterial viability, and the production of virulence factors. However, compared to the 10 nM AI-2 group, higher concentrations of AI-2 (100 nM and 1 μM) reduced biofilm formation, bacterial viability, and the production of virulence factors. Consistent with the changes in morphology, gene expression analysis revealed that AI-2 up-regulated the expression of quorum sensing-associated genes and genes encoding virulence factors at lower concentrations and down-regulated these genes at higher concentrations.

**Conclusions:**

Our study demonstrated that exogenous AI-2 acted in a dose-dependent manner to regulate *P. aeruginosa* biofilm formation and virulence factors secretion via modulating the expression of quorum sensing-associated genes and may be targeted to treat *P. aeruginosa* biofilm infections.

## Background

*Pseudomonas aeruginosa* is a well-known opportunistic pathogen associated with various acute and chronic infections in humans, especially in those who are immunocompromised. *P. aeruginosa* infections can be difficult to eradicate because *P. aeruginosa* is capable of forming biofilms, which are more resistant to physical or chemical attacks than planktonic bacteria, leading to high morbidity and mortality among infected patients [1, 2]. *P. aeruginosa* could produce a number of virulence factors, such as pyocyanin, rhamnolipids, elastase, exotoxin A, phospholipase C, and exoenzyme S, which are thought to be involved in acute or chronic infections [3].

Quorum sensing (QS) is a cell-to-cell signaling system that refers to the ability of bacteria to respond to small signaling molecules secreted by various microbial species. When the amount of QS signaling molecules accumulates to a threshold, the QS system is activated upon the identification of extracellular receptors. As typical QS signaling molecules, oligopeptides are often produced by Gram-positive bacteria, while N-acyl homoserine lactones are often produced by Gram-negative bacteria [4]. *P. aeruginosa* employs three interconnected QS systems, namely, *las*, *rhl,* and *pqs*, to control the expression of important virulence factors, and these factors play a crucial role in the development of biofilms [5]. Therefore, the QS system can be a suitable target for antimicrobial therapy. Numerous anti-infectious approaches against *P. aeruginosa* biofilms have been investigated during the past decade, such as antibiotic combinations [6] and some metal chelators exerting bactericidal and antibiofilm activities [7, 8]. However, the use of large numbers of antibiotics leads to a high prevalence of bacterial resistance, and the stability of metal chelators remains to be elucidated. Currently, chemical compounds that inhibit QS systems are being gradually investigated [9, 10].

Autoinducer-2 (AI-2) is a universal QS molecule that mediates intra- and interspecies communication. This molecule is formed from spontaneous rearrangement of 4, 5-dihydroxy-2, 3-pentanedione (DPD), which is produced by the enzyme LuxS, and is the primary QS molecule produced by many Gram-positive and Gram-negative bacteria. AI-2 has been shown to play a pivotal role in the life cycle of biofilms, including the initial bacterial aggregation and the production of virulence factors [11]. Previously, Duan et al. [12] and Roy et al. [13] found that AI-2 and AI-2 analogs had an impact on *P. aeruginosa* virulence, but the mechanism is not clear. Because *P. aeruginosa* is unable to produce AI-2 [12], the molecule might act as a parainducer, which can be sensed by the bacteria and thus affect its function. An example is reported in the study conducted by Geier et al., where AI-2 increased biofilm formation by *Mycobacterium avium*, which also cannot produce AI-2 [14].

In addition, we found high constituent ratios of *Klebsiella* spp. and *Streptococcus* spp. in the tracheal aspirates of ventilator-associated pneumonia (VAP) neonates [15], and *P. aeruginosa* is a common cause of VAP. Thus, we speculated that these AI-2 producers may facilitate biofilm formation by *P. aeruginosa*. In this study, we added different concentrations of synthetic AI-2 to *P. aeruginosa* PAO1 and evaluated biofilm formation and the production of virulence factors, with an emphasis on the underlying mechanisms of AI-2 using transcriptional analysis.

## Methods

### Bacterial strains and culture conditions

*P. aeruginosa* wild-type PAO1 was kindly provided by Professor Li Shen (Institute of Molecular Cell and Biology, New Orleans, LA, USA). It was routinely grown and maintained on Luria–Bertani (LB) plates or in LB broth at 37 °C with agitation (200 rpm). Chemically synthesized AI-2 precursor DPD [(S)-4, 5-dihydroxy-2, 3-pentanedione] was purchased from Omm Scientific (Dallas, TX, USA).

### Growth assays

Growth of PAO1 in the presence of 0.1 nM, 1 nM, 10 nM, 100 nM, and 1 μM AI-2 was measured at 600 nm at intervals of 2 h up to 24 h with a spectrophotometer (UV-1800, Shimadzu, Tokyo, Japan) [16]. All experiments were performed three times independently.

### Biofilm formation assay

A static biofilm formation assay was performed in 96-well polystyrene microtiter plates as previously described with slight modifications [17]. In brief, cells from overnight cultures were standardized to an optical density at 600 nm (OD600) of 0.05. Two hundred microliters of the diluted cultures and various concentrations of AI-2 were added to 96-well microtiter plates (Costar, USA). After incubation for 24 h at 37 °C without agitation, the medium was discarded, and the plates were gently washed three times with 200 μL phosphate-buffered saline (PBS). Then, the plates were air dried and stained with 0.1 % crystal violet for 5 min at room temperature. Unattached stain was removed, and the plates were washed three times with PBS. The bacteria-bound crystal violet was dissolved in 200 μL 95 % ethanol, and the absorbance was determined at 570 nm in the microplate reader.

### Biofilm viability

*P. aeruginosa* PAO1 cells were inoculated in LB broth at an initial OD600 of 0.05 and added to a sterile 24-well plate containing glass coverslips (Costar, USA) on which various concentrations of AI-2 were applied. Cultures were grown for 24 h without agitation at 37 °C. Coverslips were then rinsed three times with PBS and subsequently sonicated for 1 min (Tomy UD-201, Tokyo, Japan) and vortexed for 1 min at room temperature. Bacteria were harvested, enumerated by serial dilutions, and plated on LB agar. Plates were incubated at 37 °C, and bacterial counts were determined after 24 h.

### Confocal laser scanning microscopy

*P. aeruginosa* PAO1 biofilms were established in 24-well plates as mentioned earlier. Cultures were grown for 48 h without agitation at 37 °C. Coverslips were then washed and stained with SYTO9/propidium iodide according to the manufacturer’s instructions of the L13152 LIVE/DEAD BacLight bacterial viability kit (Invitrogen Molecular Probes, USA). After staining for 15 min in the dark, biofilms were washed with sterile PBS to remove the planktonic dyes and bacteria, and then biofilms were visualized by excitation with an argon laser at 488 nm (emission: 515 nm) and 543 nm (emission: 600 nm) under a Nikon A1R laser confocal microscope (Nikon, Tokyo, Japan). Live bacteria were stained green while dead bacteria were stained red.

### Virulence factor assays

For the pyocyanin assay, overnight cultures were standardized to an OD600 of 0.5 and diluted 1:10 in pyocyanin production broth (PPB; 2 % proteose peptone [Oxoid, UK], 1 % K_2_SO_4_, 0.3 % MgCl_2_ · 6H_2_O) after growth in LB medium. A 5-mL sample of diluted culture with various concentrations of AI-2 was grown in PPB for 24 h and then extracted with 3 mL chloroform. The blue layer was re-extracted into 1 mL 0.2 M HCl, yielding a red solution. The absorbance was measured at 520 nm, and the pyocyanin concentration was determined by multiplying this measurement by 17.07 [18]. Elastase activity was measured using the elastin-Congo red (ECR) assay as previously described with moderate modifications [16]. Briefly, overnight cultures were standardized to an OD600 of 0.5 and diluted 1:10 in peptone tryptic soy broth (PTSB; 5 % peptone, 0.1 % tryptic soy broth) after growth in LB medium. A 5-mL sample of diluted cultures with and without various concentrations of AI-2 was grown in PTSB for 6 h, and 100 μL filtered supernatant was added to 5-mL tubes containing 10 mg of ECR (Sigma, USA), 900 μL 10 mM Tris HCl (pH 7.5), and 1 mM CaCl_2_. The tubes were incubated for 4 h at 37 °C with shaking (250 rpm), followed by centrifugation to remove unreacted substrate. The absorbance at 495 nm was measured.

### RNA extraction and quantitative real-time PCR (qRT-PCR)

Overnight cultures of PAO1 at an initial OD600 of 0.05 were washed and then inoculated into fresh LB medium supplemented with various concentrations of AI-2 (0.1 nM to 1 μM) at an initial OD600 of 0.05. Cultures were grown at 37 °C with agitation for 24 h. Total RNA was extracted and purified using the TaKaRa Minibest Universal RNA Extraction Kit (TaKaRa, Japan) according to the manufacturer’s instructions. The concentration and purity of extracted total RNA was determined by ultraviolet absorption (260/280 nm) using a NanoDrop ND-1000 spectrophotometer (NanoDrop Technologies, Wilmington, DE, USA). The first-strand cDNA was generated from a purified mRNA sample using a PrimeScript RT reagent Kit with gDNA Eraser (TaKaRa). Real-time PCR was carried out using the SsoFast Evagreen Supermix Kit (Bio-Rad, CA, USA) with a Bio-Rad Real-Time PCR instrument. The reaction procedure was performed as follows: 95 °C for 30 s, 40 cycles of 95 °C for 5 s, and 60 °C for 5 s, and a final melting curve analysis from 65 °C to 95 °C, with increments of 0.5 °C every 5 s. Real-time PCR amplifications were conducted in triplicate.

Primer sequences for *P. aeruginosa* QS genes and virulence genes were used as described previously (Table 1). The ribosomal gene *rpsL* was chosen as a housekeeping gene to normalize the qRT-PCR data and to calculate the relative fold changes in gene expression. Amplification profiles were analyzed using Bio-Rad Manager Software, and cycle threshold (Ct) values for each target gene were normalized to the geometric mean of the Ct of *rpsL* amplified from the corresponding sample. The fold change of target genes for each group with respect to the control group was calculated using the ΔΔCt method.

**Table 1:** PCR primers for real-time RT-PCR

Gene	Primer direction	Sequence(5’-3’)	Amplicon size (bp)
*lasI*	Forward	GGCTGGGACGTTAGTGTCAT	104
	Reverse	AAAACCTGGGCTTCAGGAGT	
*lasR*	Forward	ACGCTCAAGTGGAAAATTGG	111
	Reverse	TCGTAGTCCTGGCTGTCCTT	
*rhlI*	Forward	AAGGACGTCTTCGCCTACCT	130
	Reverse	GCAGGCTGGACCAGAATATC	
*rhlR*	Forward	CATCCGATGCTGATGTCCAACC	101
	Reverse	ATGATGGCGATTTCCCCGGAAC	
*lasA*	Forward	GCGCGACAAGAGCGAATAC	94
	Reverse	CGGCCCGGATTGCAT	
*lasB*	Forward	AGACCGAGAATGACAAAGTGGAA	81
	Reverse	GGTAGGAGACGTTGTAGACCAGTTG	
*phzH*	Forward	TGCGCGAGT TCAGCCACCTG	214
	Reverse	TCCGGGACATAGTCGGCGCA	
*rhlA*	Forward	TGGCCGAACATTTCAACGT	107
	Reverse	GATTTCCACCTCGTCGTCCTT	
*rpsL*	Forward	GCAACTATCAACCAGCTGGTG	231
	Reverse	GCTGTGCTCTTGCAGGTTGTG	

### Statistical analysis

Continuous data from this study were expressed as means ± standard deviation. Independent unpaired data were analyzed using the Student’s *t*-test. One-way analysis of variance was used for multi-group comparisons. Statistical analyses were performed using SPSS version 17.0 (SPSS, Inc., Chicago, IL, USA). *P* < 0.05 was considered to be statistically significant.

## Results

### Effects of AI-2 on *P. aeruginosa* growth

**Fig. 1:**
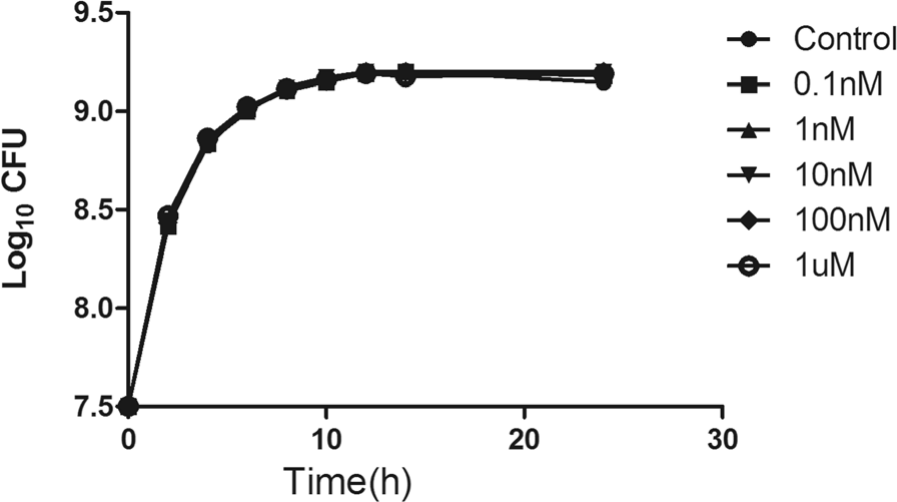
Effects of AI-2 on planktonic growth of *P. aeruginosa* PAO1. Cells were grown in LB medium, in the presence of different concentrations of AI-2 (0.1nM, 1nM, 10nM, 100nM and 1 μM). The data represent mean values of three independent experiments. Error bars represent the standard errors of the means

To test the impact of AI-2 on *P. aeruginosa* biofilm formation and virulence, we first investigated its effect on planktonic bacterial growth. The 1-μM concentration of AI-2 did not influence the growth of the planktonic cultures (Fig. 1).

### Effects of AI-2 on biofilm formation

**Fig. 2:**
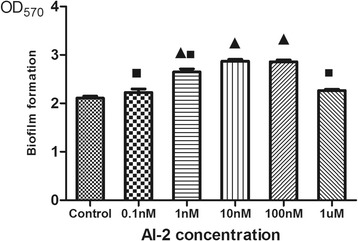
Effects of AI-2 on *P. aeruginosa* PAO1 biofilm formation. Biofilm formation was assessed by crystal violet after static incubation at 37 °C for 24 h. Error bars represent SEM and all experiments were performed in triplicate with three independent assays; Triangles denote a statistically significant difference from the control (*P* < 0.05). Squares denote a statistically significant difference from the 10nM AI-2 group (*P* < 0.05)

A dose-dependent effect of AI-2 on the biofilm formation of *P. aeruginosa* PAO1 was observed as demonstrated in Fig. 2. Biofilm formation increased in the presence of 0.1 nM, 1 nM, and 10 nM AI-2 with a 1.1-, 1.3-, and 1.4-fold increase in biofilm biomass compared to the negative control. It should be noted that 10 nM AI-2 had the greatest impact on *P. aeruginosa* PAO1 biofilm formation (*P* < 0.05). However, higher concentrations (100 nM and 1 μM AI-2) resulted in a lower biofilm biomass increase than 10 nM AI-2.

**Fig. 3:**
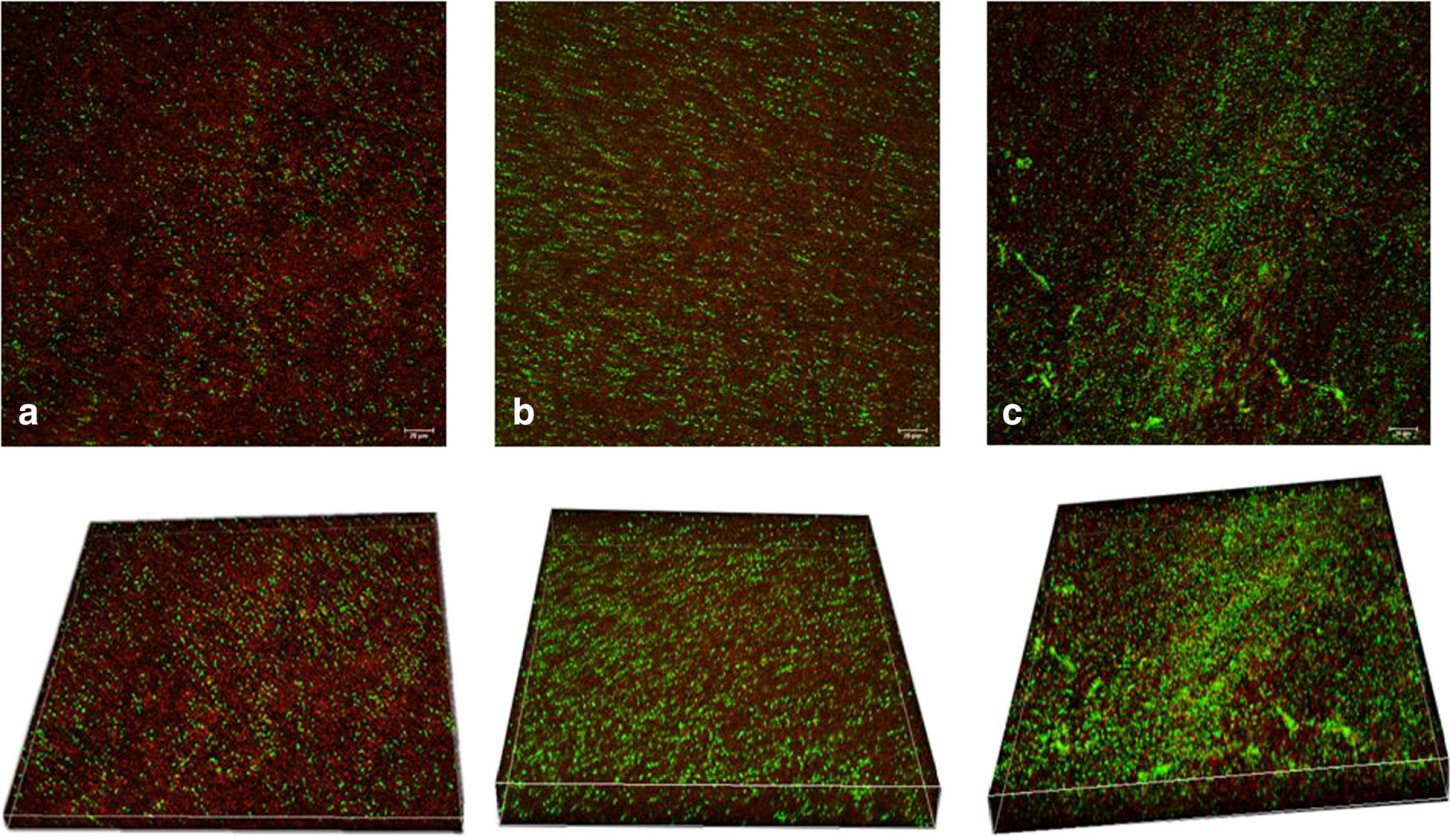
Confocal laser scanning micrographs of 2-day *P. aeruginosa* PAO1 biofilms treated under different concentrations of AI-2 (×400). Bacterial viability was determined using L13152 LIVE/DEAD BacLight bacterial viability kit. **a** No exposure to AI-2; **b** Exposure to 1nM AI-2; **c** Exposure to 10nM AI-2. Cells staining red are considered dead while cells staining green are viable cells. The scale bar represents 20 μm

**Fig. 4:**
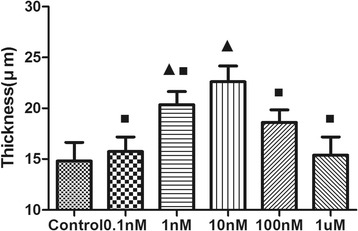
Comparison of biofilm thickness under different concentrations of AI-2. Data represent the average of three image stacks collected from randomly selected areas. Triangles denote a statistically significant difference from the control (*P* < 0.05). Squares denote a statistically significant difference from the 10nM AI-2 group (*P* < 0.05)

Consistent findings were also demonstrated by confocal laser scanning microscopy. Increased AI-2 concentrations led to increased biofilm formation and the promotion of the three-dimensional structure of the biofilm (Fig. 3). A dense and compact biofilm was observed in the 10 nM AI-2 group, and the number of viable bacteria in the control was less than that in the 1 nM and 10 nM AI-2 groups. Furthermore, the biofilm thickness in the 1 nM and 10 nM AI-2 groups was significantly increased compared with that in the control group (Fig. 4).

### Biofilm viability

**Fig. 5:**
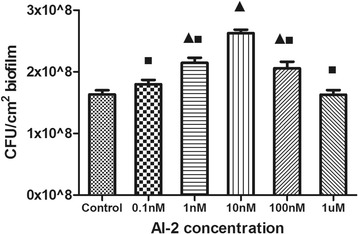
Enumeration of viable bacteria under different concentrations of AI-2. Data represent the means and standard deviations of three independent experiments. Triangles denote a statistically significant difference from the control (*P* < 0.05). Squares denote a statistically significant difference from the 10nM AI-2 group (*P* < 0.05)

The mean number of bacteria recovered from the biofilms (Fig. 5) in the 1 nM AI-2 group (2.16 × 10^8^ cfu/cm^2^), 10 nM AI-2 group (2.64 × 10^8^ cfu/cm^2^), and 100 nM AI-2 group (2.05 × 10^8^ cfu/cm^2^) was significantly greater than that in the control group (1.62 × 10^8^ cfu/cm^2^) (*P* < 0.05). However, the mean number of bacteria in the 0.1 nM AI-2 group (1.8 × 10^8^ cfu/cm^2^) and the 1 μM AI-2 group (1.66 × 10^8^ cfu/cm^2^) was slightly greater than that in the control group, but the increase was not significant (*P* > 0.05). These results were consistent with biofilm morphology changes and suggest that AI-2 increases the viability of *P. aeruginosa* PAO1 biofilms.

### Induction of virulence factor production

**Fig. 6:**
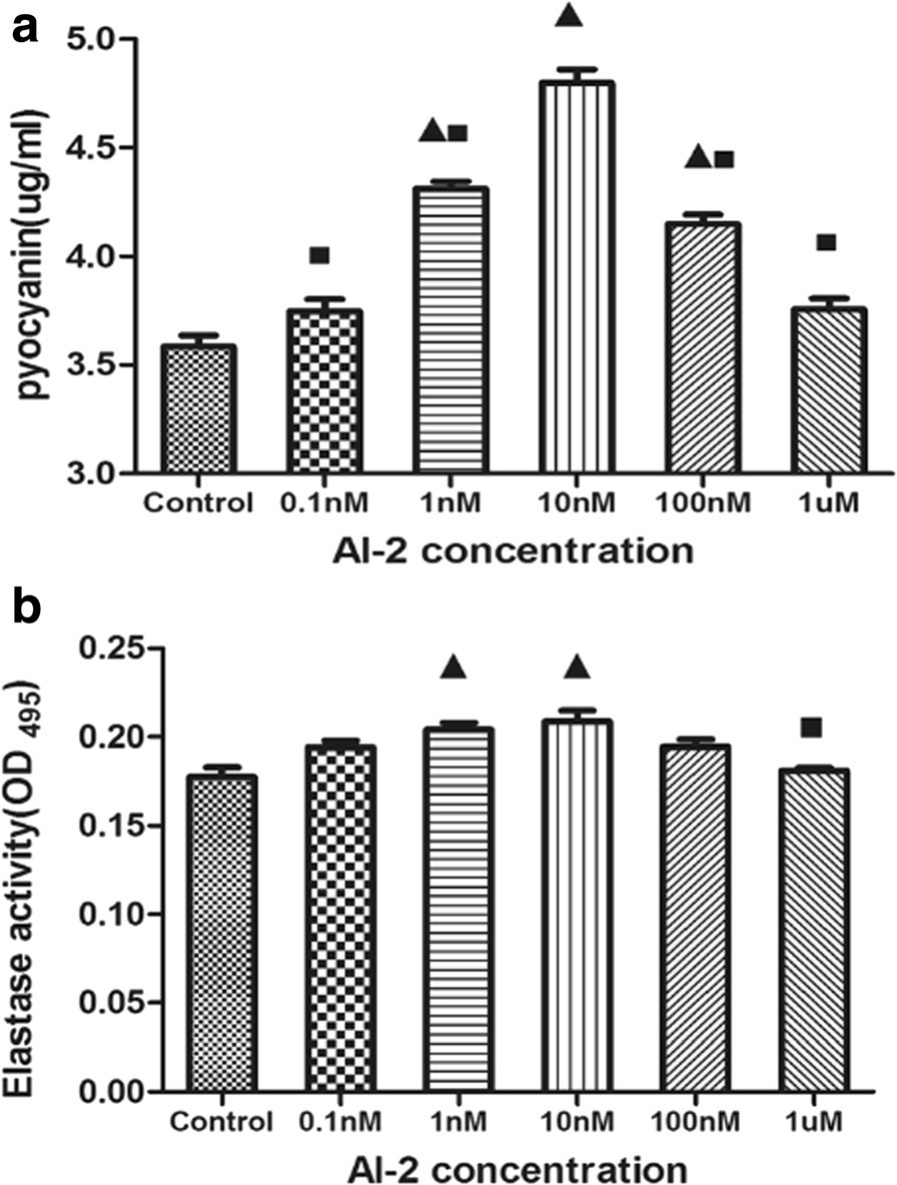
Effects of AI-2 on the production of virulence factors of *Pnn aeruginosa* PAO1. **a** Relative productions of virulence factors of pyocyanin. **b** Elastase activity. Triangles denote a statistically significant difference from the control (*P* < 0.05). Squares denote a statistically significant difference from the 10nM AI-2 group (*P* < 0.05)

To study the impact of AI-2 on *P. aeruginosa* virulence, two important factors, namely pyocyanin and elastase, which are controlled by QS were measured [19]. The activity of both pyocyanin and elastase in PAO1 were increased by AI-2 in a dose-dependent manner (Fig. 6a and b). A significant increase (*P* < 0.05) in pyocyanin and elastase production was observed in the presence of 1 nM and 10 nM AI-2.

### Gene expression analysis with qRT-PCR

QS is the most important regulator of biofilm formation by *P. aeruginosa.* To investigate whether the effect of AI-2 on the virulence of *P. aeruginosa* was the result of interference with QS, qRT-PCR was used to monitor the expression of QS-associated genes. The mRNA level of QS genes and virulence genes of *P. aeruginosa* biofilms, including *rhlI*, *rhlR*, *lasI*, *lasR* (QS-associated genes), *lasA* (encoding protease), *lasB* (encoding elastase), *phzH* (encoding pyocyanin), and *rhlA* (encoding rhamnosyltransferase), increased with increasing AI-2 concentrations (from 0 to 10 nM) and decreased with 100 nM and 1 μM AI-2 (Table 2), which was consistent with the morphology changes. Especially, with 10 nM AI-2, the expression of *lasI*, *lasR*, *rhlI*, *rhlR*, *lasA*, *lasB*, *phzH*, and *rhlA* was increased by 2.3-, 1.1-, 1.3-, 2.5-, 10-, 11-, 9.5-, and 3.7-fold, respectively; these increases were statistically significant when normalized to a control gene (*P* < 0.05). These results indicated that exogenous AI-2 could up-regulate the expression of QS-associated genes of *P. aeruginosa* PAO1.

**Table 2:** QS and virulence genes regulated by AI-2 of *P. aeruginosa* biofilm

Gene	Fold change in expression					
	0	0.1 nM	1 nM	10 nM	100 nM	1 μM
*lasI*	1	1.9 ± 0.1	2.7 ± 0.2*	3.3 ± 0.2*	2.2 ± 0.18*	2 ± 0.2*
*lasR*	1	1.3 ± 0.1	1.9 ± 0.16	2.1 ± 0.23*	1.3 ± 0.16	1.3 ± 0.2
*rhlI*	1	1.5 ± 0.17	2.1 ± 0.1*	2.3 ± 0.13*	1.7 ± 0.16	1.2 ± 0.1
*rhlR*	1	2.1 ± 0.2*	3.4 ± 0.3*	3.5 ± 0.24*	2.2 ± 0.13*	1.8 ± 0.3
*lasA*	1	4.3 ± 0.16*	5.1 ± 0.27*	11 ± 0.21*	4 ± 0.21*	3 ± 0.18*
*lasB*	1	4 ± 0.14*	5.8 ± 0.45*	12.7 ± 1.7*	5 ± 0.15*	2.8 ± 0.13*
*phzH*	1	3.6 ± 0.34*	5.8 ± 0.67*	10.6 ± 0.7*	3.8 ± 0.16*	3.3 ± 0.22*
*rhlA*	1	2.2 ± 0.16*	3.7 ± 0.33*	4.8 ± 0.56*	2.3 ± 0.14*	2.1 + 0.14*

Values marked with an asterisk (*) indicate that the fold change of relative gene expression level of *P. aeruginosa* PAO1 in the presence of different concentrations of AI-2 was significantly different from negative control at *P* < 0.05

## Discussion

*P. aeruginosa* is a prevalent environmental bacterium that is responsible for various recalcitrant infections in humans. It is also one of the most prevalent isolates in sputum samples of neonates with VAP [20]. In this study, we demonstrated that AI-2 induced virulence factor production, biofilm biomass, and bacterial viability of *P. aeruginosa* PAO1 in a dose-dependent manner, and AI-2 did not impact its planktonic growth. Furthermore, AI-2 influenced the expression of QS-associated genes (e.g., *lasI*, *lasR*, *rhlI*, and *rhlR*). This indicated that AI-2 affected the virulence of *P. aeruginosa* by inducing the activity of the QS systems.

Although the role of AI-2 as a general bacterial signaling molecule is yet to be completely unraveled, AI-2 is known to be involved in biofilm formation. AI-2 inhibits biofilm formation in *Bacillus cereus* [21], *Candida albicans* [22], and *Eikenella corrodens* [23], and it promotes biofilm formation in *Escherichia coli* [24], *Streptococcus mutans* [25], and multispecies biofilms between the two oral bacteria *Streptococcus gordonii* and *Porphyromonas gingivalis* [26]. The addition of exogenous AI-2 to *P. aeruginosa* biofilms increased biofilm formation, indicating that *P. aeruginosa* responds to the molecule. This study revealed that AI-2 can be a parainducer as a QS molecule regulating *P. aeruginosa* biofilms.

A similar concentration-dependent effect of AI-2 on biofilm formation has been reported for *Streptococcus suis* [27], *Bacillus cereus* [21], *Streptococcus oralis* [28], and *Mycobacterium avium* [14]. A previous study by Duan et al. [12] demonstrated that AI-2 was detected in sputum samples from patients with cystic fibrosis, and AI-2 regulated gene expression patterns and pathogenesis of *P. aeruginosa*. However, the mechanism is not known. In addition, Duan et al. found that the important virulence genes *lasA* and exotoxin genes *exoS* and *exoY* were not regulated by AI-2. This phenomenon may be attributed to the too high or too low concentration of AI-2 because our results showed that the *lasA* gene was affected by AI-2. Furthermore, we found that *in vitro* co-culture of the AI-2 producer *Streptococcus mitis* and *P. aeruginosa* PAO1 promoted *P. aeruginosa* PAO1 biofilm formation at certain concentrations (data not shown). It also has been suggested that the LuxS enzyme regulates metabolic processes in a large range of bacteria [29, 30]. In the present study, we found that AI-2 regulated the metabolic rate of cells in *P. aeruginosa* PAO1 biofilms. First, more viable cells were observed in the AI-2 group by confocal laser scanning microscopy, and plating experiments revealed that the bacterial reproduction of the AI-2 group is faster than that of the control group. Second, the *rhl* QS system is a metabolic regulator for *P. aeruginosa* [31]. In this study, the increased expression levels of the *rhlI* and *rhlR* genes, which are related to bacterial metabolism, may lead to increased biofilm formation and metabolic rate as the concentration of AI-2 increased.

AI-2 is a cell-signaling regulator of *P. aeruginosa*. It contributes substantially to the biofilm formation of *P. aeruginosa* and plays an important role in the pathogenesis of *P. aeruginosa* infections. This phenomenon may be due to the up-regulation of QS genes and virulence factor genes such as *lasB*, *lasA*, and *phzH*, which mediate the production of virulence factors. In fact, up-regulated transcription of autoinducer synthase (*lasI* and *rhlI*) and their cognate receptor (*lasR* and *rhlR*) genes may be responsible for the induction of PAO1 biofilm formation and secretion of elastase and pyocyanin because QS genes also mediate virulence factor genes.

## Conclusions

Taken together, this study demonstrated that AI-2 increased *P. aeruginosa* PAO1 biofilm formation, bacterial viability, and virulence production in a dose-dependent manner. Possible mechanisms responsible for the effect of AI-2 may involve the up-regulation of QS systems. Our results support the significance of intercellular signaling in bacterial survival strategies and emerging views on interference with bacterial signaling as a novel means of combating *P. aeruginosa* infections.

## Abbreviations

AI-2: Autoinducer-2
AHL: Acyl homoserine lactones
DPD: 4, 5-dihydroxy-2, 3-pentanedione
ECR: Elastin-Congo red
LB: Luria-Bertani
PBS: Phosphate buffered saline
PI: Propidium iodide
PPB: Pyocyanin production broth
QS: Quorum sensing
